# The Impact of Artificial Intelligence on BI-RADS Classification and Diagnostic Confidence in Mammography Interpretation by Radiology Residents

**DOI:** 10.3390/life16061020

**Published:** 2026-06-17

**Authors:** Ioana-Andreea Cîrlig, Alexandru-Marian Olaru, Mihai-Alexandru Ene, Aurelia-Ștefania Domenco, Rossy-Vlăduț Teică, Cristina-Mihaela Ciofiac, Raluca-Elena Nica, Violeta-Maria Novac, Mădălin Mămuleanu, Lucian-Mihai Florescu, Ioana-Andreea Gheonea

**Affiliations:** 1 Doctoral School, University of Medicine and Pharmacy of Craiova, 200349 Craiova, Romania; andreea.cirlig97@gmail.com (I.-A.C.); mihai.ene@umfcv.ro (M.-A.E.); aurelia.domenco@umfcv.ro (A.-Ș.D.); cristina.ciofiac@umfcv.ro (C.-M.C.); 2 Department of Radiology and Medical Imaging, Emergency Clinical County Hospital of Craiova, 200642 Craiova, Romania; alex.olaru89@yahoo.com; 3 Department of Radiology and Medical Imaging, University of Medicine and Pharmacy of Craiova, 200349 Craiova, Romania; rossy.teica@gmail.com (R.-V.T.); raluca.elena.nica@gmail.com (R.-E.N.); ioana.gheonea@umfcv.ro (I.-A.G.); 4 Filantropia Municipal Clinical Hospital, 200143 Craiova, Romania; mariavioletanovac@gmail.com; 5 Department of Automatic Control and Electronics, University of Craiova, 200585 Craiova, Romania; madalin.mamuleanu@edu.ucv.ro

**Keywords:** artificial intelligence, mammography, BI-RADS, radiology residents, diagnostic confidence, breast imaging, digital breast tomosynthesis, decision support, reader performance, medical education

## Abstract

Background: Artificial intelligence (AI) is increasingly used as a decision-support tool in mammography, but its influence on radiology residents’ interpretive behavior remains insufficiently characterized. This study evaluated the impact of AI assistance on BI-RADS classification and diagnostic confidence among radiology residents. Methods: This retrospective, single-center, multi-reader paired study included 112 diagnostic mammography examinations, corresponding to 223 assessable breasts and 2230 resident-breast readings. Ten radiology residents interpreted 2D mammography and digital breast tomosynthesis examinations first without AI assistance and subsequently with access to AI output. Changes in the BI-RADS category, diagnostic confidence, reasons for modification, and agreement with an expert-consensus BI-RADS reference standard were analyzed. Results: AI-assisted reassessment changed the BI-RADS classification in 9.7% of readings and diagnostic confidence in 19.2%, with any AI-associated modification observed in 24.3% of instances. Upgrades were more frequent than downgrades, particularly for medium- and high-suspicion AI outputs. Confidence increased more often than it decreased. Expert-reference agreement improved modestly, and BI-RADS 4+ sensitivity increased from 72.0% to 82.3%, with stable negative agreement, but these metrics reflect agreement with expert BI-RADS consensus rather than pathology-confirmed cancer detection. Conclusions: AI assistance influenced both BI-RADS reassessment and diagnostic confidence among radiology residents, producing modest but directionally favorable changes. These findings support cautious, supervised integration of AI into breast imaging training, with attention to confidence calibration and potential overreliance.

## 1. Introduction

Mammography interpretation remains vulnerable to inter-reader variability, even among trained radiologists [1,2]. This variability is particularly relevant in radiology residency, where trainees are still developing pattern-recognition skills, diagnostic thresholds, and confidence in breast imaging interpretation. For residents, mammography is often perceived as challenging and highly specialized, requiring dedicated expertise and repeated exposure to achieve reliable performance [3]. Previous studies have shown that interpretive accuracy improves with experience, supporting the existence of a substantial learning curve in mammographic diagnosis [4,5]. Because BI-RADS categories are directly linked to management recommendations, differences in interpretation may translate into different follow-up strategies, biopsy decisions, or reassurance for the same imaging findings. Therefore, decision-support tools capable of improving consistency, supporting less experienced readers, and reducing interpretive variability are increasingly relevant in clinical practice [6,7].

Artificial intelligence (AI) has rapidly emerged as a promising adjunct in mammography interpretation. Several studies have demonstrated that AI-based systems can improve diagnostic performance, support lesion detection, and increase inter-reader agreement [8,9,10,11]. AI-assisted interpretation has also been associated with improved BI-RADS classification accuracy among radiologists [12]. However, most available evidence has focused primarily on diagnostic performance metrics or on experienced readers. Considerably less is known about how AI influences less experienced readers, particularly radiology residents, who may be more susceptible both to educational benefit and to overreliance on algorithmic output. Moreover, the effect of AI extends beyond accuracy alone. In training environments, AI may influence how residents revise BI-RADS classifications, how confident they become in their assessments, and whether changes are driven by true image re-evaluation or by trust in the AI system itself.

The aim of this study was to evaluate the impact of AI assistance on mammography interpretation by radiology residents. Specifically, we assessed changes in BI-RADS classification and diagnostic confidence between unaided and AI-assisted readings, as well as the reported reasons underlying these changes. In a secondary expert-reference analysis, we evaluated whether AI-assisted reassessment moved resident BI-RADS interpretations closer to or farther from an expert-consensus standard. By combining BI-RADS transitions, confidence changes, reader-reported reasons for modification, and expert-reference comparison, this study aimed to characterize how AI output is associated with decision-making behavior during reassessment in a radiology training setting.

## 2. Materials and Methods

### 2.1. Study Design

This study was designed as a retrospective, single-center, multi-reader paired investigation evaluating changes in mammography interpretation during AI-assisted reassessment by radiology residents. Each resident assessed the same mammographic examinations in two sequential reading sessions: first without AI assistance, and subsequently with access to AI output. The primary behavioral outcome was modification of BI-RADS classification during AI-assisted reassessment. Secondary outcomes included changes in diagnostic confidence, direction of BI-RADS modification, confidence-only changes, reader-reported reasons for modification, and the perceived added or misleading value of AI. Because there was no AI-free second-read control arm and the unaided session always preceded the AI-assisted reassessment session, the design cannot isolate the independent causal effect of AI from repeat reading, temporal learning, recall, or anchoring to the initial assessment.

A secondary expert-reference analysis was performed using consensus-based BI-RADS classifications established by two expert breast radiologists, each with more than 10 years of experience in breast imaging. The experts independently reviewed all examinations and assigned BI-RADS reference categories while blinded to resident assessments, AI-assisted modifications, and questionnaire responses. Disagreements were resolved by consensus during a joint review session, resulting in a final expert-consensus BI-RADS reference standard. This analysis assessed whether AI-assisted reassessment moved resident interpretations closer to, farther from, or left them unchanged relative to the expert-consensus standard. The reference standard was used to evaluate agreement with expert BI-RADS assessment and was not intended to define pathology-confirmed disease status.

### 2.2. Study Population and Case Selection

A total of 112 consecutive diagnostic mammography examinations performed at a single academic imaging center between March and September 2025 were retrospectively included. Eligible examinations consisted of diagnostic mammography studies with available full-field digital mammography and digital breast tomosynthesis datasets, as well as complete AI output for analysis.

The cohort included 59 examinations performed in asymptomatic patients, whereas the remaining cases were referred for clinical symptoms or for problem-solving assessment following abnormalities detected on breast ultrasound. Examinations performed for local staging of previously diagnosed breast cancer were excluded, in order to avoid inclusion of cases in which the presence and location of malignancy were already established before mammographic interpretation.

Patient age ranged from 25 to 81 years. Breast density was recorded according to the BI-RADS classification. Among the 112 examinations, 45 were classified as density category A, 43 as category B, 20 as category C, and 4 as category D.

In line with the study design, each examination was analyzed at the breast level, with separate assessments performed for the right and left breasts. BI-RADS classification, diagnostic confidence, and AI output were therefore recorded independently for each breast. One right breast was excluded because of prior mastectomy, resulting in 223 assessable case-breasts. Each assessable case-breast was evaluated by 10 radiology residents, yielding a final analytic dataset of 2230 resident-breast readings.

### 2.3. Image Acquisition Protocol

All mammographic examinations were acquired using a Fujifilm Amulet Innovality digital mammography system (Fujifilm, Tokyo, Japan) according to standard institutional diagnostic breast imaging protocols. Each examination included full-field digital mammography (2D) and digital breast tomosynthesis (DBT), with standard craniocaudal (CC) and mediolateral oblique (MLO) views obtained for each breast when technically feasible.

### 2.4. AI System

An AI-based decision-support system, BreastView 1.1.0 (Gleamer, Paris, France), was used in this study. The system analyzes full-field digital mammography images and provides AI-generated suspicion categories based on detected imaging features. For each case, the AI output was classified as no findings, low suspicion, medium suspicion, or high suspicion. In addition, the software provides visual markers highlighting regions of interest corresponding to suspected abnormalities.

The AI analysis was based exclusively on 2D mammography images. In contrast, residents had access to both 2D mammography and DBT datasets during image interpretation. Therefore, the study evaluated the influence of AI output generated from 2D mammography when added to a resident interpretation workflow that included both 2D mammography and DBT.

### 2.5. Readers

Ten radiology residents participated as readers. All readers had prior exposure to breast imaging and were familiar with the BI-RADS classification system. Their experience in senology ranged from 3 to 12 months, reflecting different stages of breast imaging training within the radiology residency program.

For subgroup analysis, residents were categorized into two experience groups: five readers with ≤6 months of senology experience were classified as less experienced, whereas five readers with >6 months of experience were classified as more experienced.

Descriptive subgroup analyses were performed according to resident experience group, comparing less experienced and more experienced readers with respect to BI-RADS modification, confidence modification, any AI-associated modification, and expert-reference movement. Because experience ranged from 3 to 12 months and the number of readers was small, these subgroup analyses were considered exploratory and were not used for definitive inference.

During image interpretation, all readers were blinded to clinical information, prior imaging reports, histopathological results, follow-up data, and the expert-consensus BI-RADS reference standard. In the unaided reading session, readers were also blinded to AI output. In the AI-assisted reassessment session, the readers had access to the AI output in addition to the 2D mammography images and DBT datasets.

### 2.6. Reading Protocol

Each reader independently evaluated all examinations in two sequential reading sessions. Data were recorded separately for each case and for each breast. A washout period of four weeks was maintained between the two sessions to reduce recall bias. Consequently, the observed changes should be interpreted as reassessment behavior in an anchored AI-assisted setting rather than as independent AI-assisted interpretation.

In the first reading session, examinations were interpreted using 2D mammography and DBT datasets without access to AI assistance. For each breast, readers assigned a BI-RADS category from 1 to 5 and a diagnostic confidence score using a 5-point Likert scale, where 1 indicated the lowest confidence and 5 indicated the highest confidence.

In the second reading session, the same examinations were reassessed with access to the AI output in addition to the 2D mammography and DBT datasets. The AI suspicion category provided by the system was recorded as no findings, low suspicion, medium suspicion, or high suspicion. During this reassessment session, readers were also provided with their own initial BI-RADS classification and diagnostic confidence score from the first session and were asked to reassess each breast individually. Therefore, the study was designed to evaluate AI-assisted reassessment rather than fully independent second-read performance.

Readers were allowed to maintain or modify their initial assessment for each breast. In the event of modification, changes could involve BI-RADS classification, diagnostic confidence, or both; alternatively, readers could leave both parameters unchanged. For each breast, the final BI-RADS category and final confidence score assigned during the AI-assisted session were recorded, allowing direct paired comparison with the initial unaided assessment. Changes in BI-RADS classification were categorized as upgrade, downgrade, or unchanged. Changes in diagnostic confidence were categorized as increase, decrease, or unchanged.

Whenever a modification was made in BI-RADS classification, diagnostic confidence, or both, readers were required to indicate the reason for the change using predefined categories: AI highlighted an overlooked finding, trust in AI over initial judgment, or other/unclear.

### 2.7. Derived Variables and Operational Definitions

Derived variables were created to characterize AI-associated modifications in BI-RADS classification, diagnostic confidence, and clinically relevant threshold transitions. The following derived variables were created:

- BI-RADS change: Final BI-RADS category different from initial BI-RADS category.
- BI-RADS direction: Upgrade, downgrade, or unchanged.
- BI-RADS delta: Final BI-RADS category minus initial BI-RADS category.
- Confidence change: Final confidence score different from initial confidence score.
- Confidence delta: Final confidence score minus initial confidence score.
- Confidence-only change: Confidence score changed while BI-RADS category remained unchanged.
- Any AI-associated modification: Either BI-RADS classification or diagnostic confidence changed after AI-assisted reassessment.
- Clinically relevant threshold crossing: Movement across the BI-RADS 3+ threshold or across the BI-RADS 4+ threshold.

BI-RADS 4 subcategories were not analyzed separately because the resident data were collected using BI-RADS categories 1–5 and the primary threshold analysis focused on BI-RADS 4+ as a clinically actionable suspicious-category threshold.

### 2.8. Outcome Measures

The primary outcome was the proportion of resident-breast readings in which BI-RADS classification changed after AI-assisted reassessment.

Secondary behavioral outcomes included BI-RADS upgrades, BI-RADS downgrades, BI-RADS delta, changes in diagnostic confidence, confidence increases, confidence decreases, confidence-only changes, any AI-associated modification, and clinically relevant threshold crossings at BI-RADS 3+ and BI-RADS 4+.

Reader-reported secondary outcomes consisted of the reasons for modification, recorded whenever residents changed BI-RADS classification, diagnostic confidence, or both during AI-assisted reassessment.

For the expert-reference analysis, outcomes included exact BI-RADS agreement with the expert-consensus reference standard, within-one-category BI-RADS agreement, mean absolute BI-RADS error, and AI-associated movement relative to the reference standard. AI-associated movement was classified as positive when the final post-AI BI-RADS category was closer to the expert-consensus reference than the initial BI-RADS category, negative when it was farther away, and neutral when the absolute BI-RADS error was unchanged.

Agreement with the expert-consensus BI-RADS thresholds was evaluated at BI-RADS 4+ as the primary clinical threshold and at BI-RADS 3+ as a secondary threshold. These analyses were reported as expert-threshold agreement metrics, including positive agreement, negative agreement, positive predictive agreement, negative predictive agreement, overall agreement, and balanced agreement. These metrics were calculated using the expert-consensus BI-RADS reference standard and should not be interpreted as pathology-confirmed cancer detection performance.

### 2.9. Statistical Analysis

Descriptive statistics were used to summarize patient characteristics, breast density distribution, initial and final BI-RADS distributions, diagnostic confidence scores, AI suspicion categories, BI-RADS transition patterns, direction of BI-RADS change, confidence changes, and reasons for modification. Categorical variables were presented as counts and percentages. Diagnostic confidence was analyzed as an ordinal variable ranging from 1 to 5 and was summarized using score distributions and mean changes.

The proportion of BI-RADS changes after AI-assisted reassessment was calculated as the primary behavioral endpoint. Among readings with BI-RADS modification, the observed frequency of upgrades versus downgrades was summarized descriptively. Exact binomial testing for upgrade versus downgrade direction, exact sign testing for confidence increases versus decreases, and exact McNemar testing for BI-RADS threshold crossings were retained as unadjusted exploratory analyses.

For the expert-reference analysis, resident readings were merged with the expert-consensus BI-RADS reference classifications by case identifier and breast side. Agreement with the expert-consensus reference standard was evaluated using exact BI-RADS agreement, within-one-category agreement, and mean absolute BI-RADS error. Mean absolute BI-RADS error was calculated as the absolute difference between the resident-assigned BI-RADS category and the expert-consensus BI-RADS category. AI-associated movement was classified as positive when the final post-AI BI-RADS category was closer to the expert-consensus reference than the initial BI-RADS category, negative when it was farther away, and neutral when the absolute BI-RADS error was unchanged.

Agreement with expert-consensus BI-RADS thresholds was evaluated at BI-RADS 4+ as the primary clinical threshold and at BI-RADS 3+ as a secondary threshold. Expert-threshold agreement metrics were calculated before and after AI-assisted reassessment using the expert-consensus BI-RADS reference standard. These metrics describe agreement with expert BI-RADS consensus rather than disease-level diagnostic accuracy.

Because the dataset contained repeated observations from the same readers and the same case-breasts, cluster-adjusted sensitivity analyses were performed for key comparisons using regression models with two-way cluster-robust standard errors by reader and case-breast. Binary outcomes were analyzed using logistic regression, and mean absolute BI-RADS error was analyzed using linear regression. Cluster-adjusted estimates, 95% confidence intervals, and *p*-values were used to assess the robustness of the descriptive findings.

Descriptive subgroup analyses were performed according to resident experience group, comparing less experienced and more experienced readers with respect to BI-RADS modification, confidence modification, any AI-associated modification, and expert-reference movement.

All *p*-values were interpreted descriptively because of the exploratory reader-behavior design, the clustered data structure, and the limited number of readers. Emphasis was placed on observed effect sizes, 95% confidence intervals, and consistency across complementary outcomes rather than on dichotomous statistical significance.

All analyses were performed using Python 3.12.13, with pandas 2.2.3, numpy 2.3.5, and openpyxl 3.1.5. The analysis code and cleaned dataset were generated reproducibly from the original resident-level data files.

### 2.10. Ethics Approval

This retrospective study was approved by the institutional ethics committee of the University of Medicine and Pharmacy of Craiova, Romania (Approval No. 72/21.04.2026). The approval covered the retrospective use of archived mammographic examinations acquired between March and September 2025. The requirement for informed consent was waived because of the retrospective study design and the use of anonymized data.

## 3. Results

### 3.1. Dataset and Baseline Characteristics

The final analytic dataset included 10 radiology residents, 112 mammographic examinations, and 223 assessable case-breasts. One right breast was excluded because of prior mastectomy. Because each assessable case-breast was evaluated by each of the 10 residents, the final dataset comprised 2230 resident-breast readings.

Patient age ranged from 25 to 81 years. Among the 112 examinations, 59 were performed in asymptomatic patients. Breast density distribution according to the BI-RADS classification was as follows: density A in 45 examinations, density B in 43 examinations, density C in 20 examinations, and density D in 4 examinations. All residents interpreted 2D mammography and DBT datasets, whereas the AI system analyzed 2D mammography images only.

The main cohort, imaging, and reader characteristics are summarized in Table 1.

### 3.2. BI-RADS Distribution Before and After AI-Assisted Reassessment

At the initial unaided assessment, BI-RADS 2 was the most frequent category, assigned in 1243 of 2230 readings (55.7%). BI-RADS 1 was assigned in 265 readings (11.9%), BI-RADS 3 in 386 readings (17.3%), BI-RADS 4 in 254 readings (11.4%), and BI-RADS 5 in 82 readings (3.7%).

After AI-assisted reassessment, the overall BI-RADS distribution remained broadly similar, but with a modest shift toward higher BI-RADS categories, as highlighted in Table 2.

### 3.3. BI-RADS Modification After AI-Assisted Reassessment

BI-RADS classification changed after AI-assisted reassessment in 216 of 2230 resident-breast readings (9.7%). In the remaining 2014 readings (90.3%), the BI-RADS category remained unchanged.

Among all readings, 125 were upgraded (5.6%) and 91 were downgraded (4.1%) after AI-assisted reassessment. Among the 216 readings with BI-RADS modification, upgrades were numerically more frequent than downgrades (125 vs. 91). In the cluster-adjusted sensitivity analysis, the estimated probability of upgrade among changed readings was 57.9% (95% CI 39.7 to 74.1%; *p* = 0.398 for comparison with 50%). Clinically relevant threshold crossings were also observed. As highlighted in Table 3, movement from BI-RADS below 3 to BI-RADS 3 or higher occurred in 78 readings, whereas movement from BI-RADS 3 or higher to below 3 occurred in 67 readings. Movement from below BI-RADS 4 to BI-RADS 4 or higher occurred in 63 readings, whereas movement from BI-RADS 4 or higher to below 4 occurred in 33 readings. In cluster-adjusted analysis, the BI-RADS 4+ classification rate increased from 15.1% to 16.4% (OR 1.11, 95% CI 0.99 to 1.24; *p* = 0.079).

**Figure 1:**
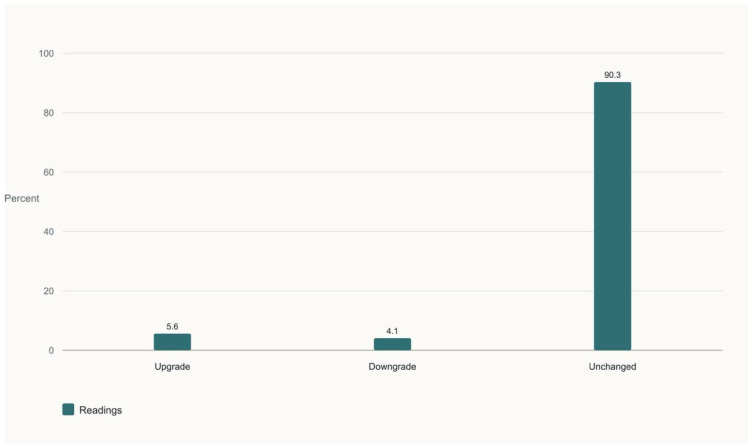
Direction of BI-RADS modification after AI-assisted reassessment. The figure shows the proportion of resident-breast readings that remained unchanged, were upgraded, or were downgraded after AI-assisted reassessment. BI-RADS classification changed in 216 of 2230 readings, including 125 upgrades and 91 downgrades.

The direction of BI-RADS modification after AI-assisted reassessment is illustrated in Figure 1.

### 3.4. Diagnostic Confidence Modification

Initial diagnostic confidence was generally high. Confidence scores of 4 or 5 were recorded in 1562 of 2230 readings (70.0%) before AI-assisted reassessment, as illustrated in Table 4. After AI-assisted reassessment, confidence scores of 4 or 5 were recorded in 1812 readings (81.3%).

Mean confidence increased from 3.95 before AI-assisted reassessment to 4.16 after AI-assisted reassessment, corresponding to a mean confidence delta of +0.21.

Diagnostic confidence changed in 429 of 2230 readings (19.2%). Confidence increased in 372 readings (16.7%) and decreased in 57 readings (2.6%), while remaining unchanged in 1801 readings (80.8%). Among readings with changed confidence, confidence increases remained more frequent than confidence decreases in the cluster-adjusted analysis (estimated probability of increase 86.7%, 95% CI 71.0 to 94.6%; *p* < 0.001 for comparison with 50%). Confidence-only change, defined as a change in confidence without a BI-RADS modification, occurred in 325 readings (14.6%).

Any AI-associated modification, defined as either BI-RADS modification or confidence modification, occurred in 541 of 2230 readings (24.3%).

Initial confidence was inversely associated with subsequent modification. BI-RADS changed in 23.3% of readings with an initial confidence of 1 and 27.0% of readings with an initial confidence of 2, compared with 3.6% of readings with an initial confidence of 5. Any AI-associated modification occurred in 76.7% of readings with an initial confidence of 1, 62.9% with a confidence of 2, 49.6% with a confidence of 3, 17.9% with a confidence of 4, and 5.3% with a confidence of 5.

### 3.5. Influence of AI Suspicion Category

AI suspicion category was associated with the frequency and direction of resident modification, as illustrated in Table 5. BI-RADS changed in 79 of 1460 readings with no AI findings (5.4%), 6 of 70 readings with low-suspicion AI findings (8.6%), 65 of 370 readings with medium-suspicion findings (17.6%), and 66 of 330 readings with high-suspicion findings (20.0%).

The direction of BI-RADS change differed according to AI suspicion category. When AI reported no findings, downgrades were more common than upgrades, with 67 downgrades and 12 upgrades. In contrast, high-suspicion AI findings were associated predominantly with upgrades, with 63 upgrades and 3 downgrades. Medium-suspicion AI findings were also mainly associated with upgrades, with 49 upgrades and 16 downgrades.

Any AI-associated modification increased with AI suspicion category. It occurred in 279 of 1460 readings with no AI findings (19.1%), 19 of 70 readings with low-suspicion findings (27.1%), 120 of 370 readings with medium-suspicion findings (32.4%), and 123 of 330 readings with high-suspicion findings (37.3%).

**Figure 2:**
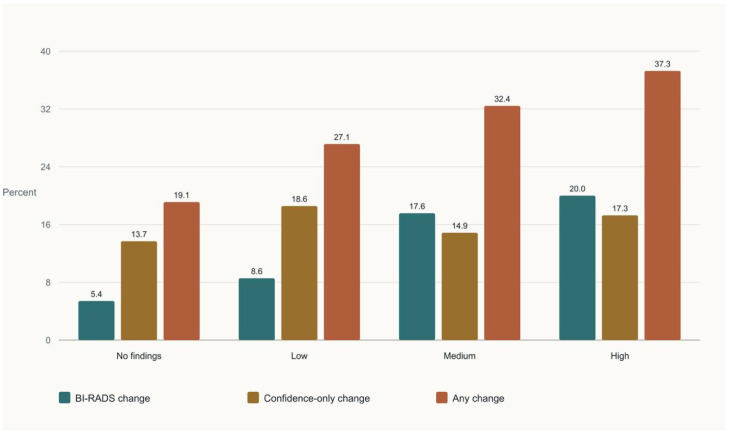
Resident modification rates according to AI suspicion category. The figure shows BI-RADS modification, confidence-only modification, and any AI-associated modification according to AI suspicion category. Modification rates increased with AI suspicion level, with the highest rates observed for medium- and high-suspicion AI outputs.

Modification rates increased across AI suspicion categories, as shown in Figure 2.

### 3.6. Reader-Reported Reasons for Modification

Among the 216 readings with BI-RADS modification, the reported reason was that AI highlighted an overlooked finding in 79 readings (36.6%), trust in AI over the initial judgment in 70 readings (32.4%), and other or unclear reasons in 67 readings (31.0%), as shown in Table 6.

The reason profile differed for confidence-only changes. Among the 325 confidence-only changes, trust in AI over the initial judgment was reported in 271 readings (83.4%), other or unclear reasons in 36 readings (11.1%), and AI highlighting an overlooked finding in 18 readings (5.5%).

When BI-RADS and confidence changes were considered together, the most common reason for any modification was trust in AI over the initial judgment, reported in 341 of 541 modified readings (63.0%). AI highlighting an overlooked finding accounted for 97 readings (17.9%), and other or unclear reasons accounted for 103 readings (19.0%).

### 3.7. Expert-Reference BI-RADS Agreement

As Table 7 illustrates, exact agreement with the expert-consensus BI-RADS reference standard increased descriptively from 46.6% before AI-assisted reassessment to 47.6% after AI-assisted reassessment. In the cluster-adjusted sensitivity analysis, this change was not statistically significant (OR 1.04, 95% CI 0.95 to 1.14; *p* = 0.399). Within-one-category agreement increased from 88.6% to 90.0%, and mean absolute BI-RADS error decreased from 0.666 to 0.636; the cluster-adjusted difference in mean absolute error was −0.030 (95% CI −0.063 to 0.003; *p* = 0.077).

AI-assisted reassessment moved resident BI-RADS classifications closer to the expert-consensus BI-RADS reference standard in 131 readings and farther from the reference standard in 80 readings. In 2019 readings, the absolute BI-RADS error remained unchanged.

Among the 216 readings in which the BI-RADS classification changed after AI-assisted reassessment, 131 changes (60.6%) moved closer to the expert-consensus reference standard, 80 changes (37.0%) moved farther away, and 5 changes (2.3%) produced no change in absolute BI-RADS error.

**Figure 3:**
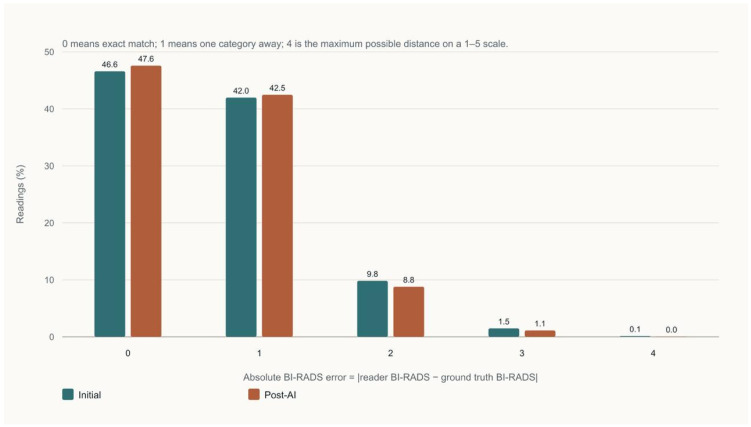
Absolute BI-RADS error before and after AI-assisted reassessment. Absolute BI-RADS error was defined as the absolute difference between the resident-assigned BI-RADS category and the expert-consensus BI-RADS reference category. An error of 0 indicates exact agreement. Mean absolute BI-RADS error decreased from 0.666 before AI-assisted reassessment to 0.636 after AI-assisted reassessment.

The distribution of absolute BI-RADS error before and after AI-assisted reassessment is shown in Figure 3.

### 3.8. Agreement with Expert-Consensus BI-RADS Thresholds

Threshold analyses were evaluated using the expert-consensus BI-RADS reference standard and therefore describe agreement with expert BI-RADS classification rather than pathology-confirmed disease detection. At the BI-RADS 4+ threshold, positive agreement with the expert-consensus reference increased from 72.0% before AI-assisted reassessment to 82.3% after AI-assisted reassessment, while negative agreement remained stable at approximately 93.8%. Overall threshold agreement increased from 90.9% to 92.3%, and balanced agreement increased from 82.9% to 88.1%.

At the BI-RADS 3+ threshold, positive agreement increased from 81.3% to 85.6%, negative agreement increased from 78.0% to 78.3%, and overall agreement increased from 78.6% to 79.6%.

Agreement with expert-consensus BI-RADS thresholds before and after AI-assisted reassessment is summarized in Table 8.

To account for repeated readings by the same residents and repeated assessment of the same case-breasts, key outcomes were re-evaluated using cluster-adjusted sensitivity analyses with two-way cluster-robust standard errors. The results are presented in Table 9. After cluster adjustment, the increase in diagnostic confidence remained robust, whereas the numerical predominance of BI-RADS upgrades and the increase in BI-RADS 4+ classification rate were attenuated.

### 3.9. Resident-Level Variability

Upgrade and downgrade patterns also varied between residents. Some readers showed a predominance of upgrades after AI-assisted reassessment, whereas others showed more frequent downgrades.

Detailed resident-level results are reported using anonymized reader identifiers in Table 10.

## 4. Discussion

### 4.1. Main Findings

This multi-reader paired study evaluated the influence of AI assistance on mammography interpretation by radiology residents, with emphasis on BI-RADS reassessment, diagnostic confidence, reader-reported reasons for modification, and agreement with an expert-consensus BI-RADS reference standard. The main finding was that AI-assisted reassessment was associated with measurable changes in resident interpretation. BI-RADS classification changed in 9.7% of resident-breast readings, while diagnostic confidence changed in 19.2%. When BI-RADS and confidence modifications were considered together, AI was associated with some form of reassessment-related modification in nearly one quarter of readings.

These findings indicate that AI did not simply act as a passive adjunct. Rather, AI output influenced both categorical decision-making and the subjective certainty with which residents approached mammographic interpretation. Importantly, the magnitude of AI influence was not uniform. Modification rates varied according to AI suspicion category, initial diagnostic confidence, and individual reader behavior. In addition, the expert-consensus analysis showed that AI-assisted reassessment was associated with a modest overall movement toward expert BI-RADS interpretation, although not all AI-associated changes were favorable. This supports the concept that AI-assisted interpretation in training environments should be understood primarily through its effects on reader behavior, confidence calibration, and reassessment patterns, while expert-reference results should be interpreted as agreement with expert BI-RADS consensus rather than disease-level diagnostic accuracy.

### 4.2. AI Influence on BI-RADS Reassessment

The observed BI-RADS modification rate of 9.7% suggests that AI-assisted reassessment affected a meaningful minority of resident interpretations. Although most BI-RADS classifications remained unchanged, AI output was associated with both the frequency and direction of resident modification. Upgrades were numerically more frequent than downgrades, and AI-assisted reassessment was associated descriptively with a modest shift toward higher BI-RADS categories, particularly BI-RADS 4 and 5. However, after accounting for clustering by reader and case-breast, the predominance of upgrades among changed readings was not statistically significant.

The direction of change was coherent with the AI suspicion category. When the AI system reported no findings, downgrades were more common than upgrades. Conversely, medium- and high-suspicion AI outputs were mainly associated with BI-RADS upgrades. This suggests that residents incorporated AI output in a directionally consistent manner: higher AI suspicion tended to increase resident suspicion, whereas absence of AI findings tended to support lower BI-RADS assessment.

These findings are consistent with previous mammography studies showing that AI can influence BI-RADS assignment, diagnostic performance, and reader agreement [8,9,10,11,12]. Dang et al. reported improved BI-RADS categorization with AI assistance [12], while Ahmadzade et al. observed improved inter- and intra-reader agreement in digital mammography with AI support [9]. Similarly, van Winkel et al. demonstrated that AI assistance may affect radiologist performance in mammography interpretation [13].

Beyond conventional mammography AI systems, additional approaches developed for BI-RADS classification have also shown promising results. Zhang et al. proposed an explainable neural network model aligned with BI-RADS morphological criteria [14], while Al Mansour et al. developed a mammography-specific architecture for BI-RADS classification [15]. More recent work has also explored large language model–based workflows for BI-RADS classification, suggesting that AI may support classification accuracy and reader performance, including among less experienced radiologists [16], although such approaches differ substantially from dedicated image-analysis systems such as the one used in the present study.

Taken together, these studies provide a useful background for understanding AI-assisted BI-RADS assessment. The present study extends this perspective by examining how radiology residents revise their own initial interpretations after AI exposure. In clinical practice and training, AI does not merely provide a final diagnostic output; it may reshape how readers assign suspicion, reconsider equivocal findings, and decide whether a case crosses a management-relevant BI-RADS threshold. Therefore, the effect of AI on BI-RADS reassessment is clinically and educationally relevant, even when the overall proportion of changed classifications is relatively modest.

### 4.3. Diagnostic Confidence and Confidence Calibration

A major finding of this study was that diagnostic confidence changed more frequently than BI-RADS classification. Confidence changed in 19.2% of readings, whereas BI-RADS classification changed in 9.7%. Moreover, confidence increased substantially more often than it decreased, and the proportion of high-confidence assessments rose after AI-assisted reassessment. This was the most robust behavioral signal after cluster-adjusted analysis.

This pattern suggests that AI frequently acted as a confirmatory influence during reassessment. In many cases, residents did not change the final BI-RADS category but became more confident after reviewing the AI output. This may be beneficial when AI reinforces a correct interpretation, particularly in early training, where uncertainty is common. However, because the study did not include an AI-free second-read control arm, the confidence increase cannot be attributed to AI alone and may also reflect repeat reading, anchoring to the initial assessment, or temporal learning. Confidence calibration is critical: an AI tool that increases confidence in correct interpretations may be educationally valuable, whereas one that increases confidence in incorrect interpretations may reinforce error.

This issue is consistent with broader concerns regarding clinician interaction with AI-based decision support. Automation bias has been described as an important risk when users place excessive trust in automated recommendations [17], and recent discussions of AI-assisted healthcare have emphasized that trust in algorithmic outputs must be calibrated rather than assumed [18,19]. In this context, the frequent confidence increases observed in our study should be interpreted as evidence that AI influenced resident certainty, not necessarily as evidence that interpretation became more accurate. This distinction is particularly relevant when AI is used by readers who are still developing stable diagnostic thresholds.

The association between initial confidence and subsequent modification further supports the importance of confidence calibration. Residents were more likely to modify BI-RADS classification or confidence when their initial confidence was low, whereas high initial confidence was associated with fewer changes. This finding is intuitive but important. It suggests that AI exerts its greatest influence precisely in cases where residents feel uncertain. From an educational perspective, these are the cases in which AI may prompt useful reconsideration. From a safety perspective, however, they are also the cases in which residents may be most vulnerable to overreliance.

Therefore, the educational objective should not be simply to increase resident confidence after AI exposure. The more appropriate goal is well-calibrated confidence: residents should become more confident when AI output is concordant with valid image-based reasoning, but remain appropriately cautious when AI output conflicts with their own assessment or when the imaging findings remain ambiguous.

### 4.4. Expert-Reference Agreement and Diagnostic Threshold Performance

After AI-assisted reassessment, residents’ BI-RADS classifications showed a slight movement toward the expert-consensus BI-RADS reference standard. Exact BI-RADS agreement increased from 46.6% to 47.6%, within-one-category agreement increased from 88.6% to 90.0%, and mean absolute BI-RADS error decreased from 0.666 to 0.636. Although these changes were numerically modest, they were directionally favorable, suggesting that AI-assisted reassessment slightly improved alignment between resident interpretations and expert BI-RADS assessment.

More importantly, among readings in which BI-RADS classification changed after AI-assisted reassessment, 60.6% moved closer to the expert-consensus reference standard, whereas 37.0% moved farther away. This finding is central to interpreting the expert-reference analysis. AI assistance was not uniformly corrective: although more changes moved residents toward expert interpretation than away from it, a relevant proportion of AI-associated changes were unfavorable. This supports a balanced interpretation of AI assistance as a tool that may improve some resident decisions while also introducing the possibility of misdirection.

The most clinically relevant signal was observed at the BI-RADS 4+ threshold. Sensitivity increased from 72.0% before AI-assisted reassessment to 82.3% after AI-assisted reassessment, while specificity remained stable at approximately 93.8%. Balanced accuracy also increased from 82.9% to 88.1%. Because BI-RADS 4+ is closely linked to clinical escalation and biopsy consideration, improved agreement with the expert-consensus BI-RADS 4+ threshold may represent the most meaningful expert-reference finding in this study.

These findings are broadly consistent with prior studies suggesting that AI assistance may improve diagnostic performance and BI-RADS assignment in mammography. Rodríguez-Ruiz et al. reported that AI-supported mammography interpretation improved reader performance compared with unaided reading [20], while Dang et al. found that AI assistance improved correct BI-RADS assignment without increasing interpretation time [12]. In the present study, the favorable movement at the BI-RADS 4+ threshold suggests that AI-assisted reassessment may help residents align more closely with expert-level suspicion thresholds. However, our results differ from many performance-focused studies because the reference standard was expert-consensus BI-RADS classification rather than pathology-confirmed disease status.

Therefore, the observed improvement should be interpreted as improved agreement with the expert-consensus BI-RADS assessment, particularly at a clinically relevant threshold, rather than as definitive evidence of improved cancer detection. This distinction is methodologically important and should be maintained throughout the manuscript.

### 4.5. Automation Bias and Resident–AI Interaction

The reader-reported reasons for modification provide insight into how residents interacted with AI output during reassessment. Among BI-RADS changes, the reasons were relatively balanced: AI highlighted an overlooked finding, residents placed greater trust in AI than in their initial judgment, or the reason was other or unclear. This suggests that BI-RADS modifications were not driven by a single mechanism. In some cases, AI may have prompted active image re-evaluation by drawing attention to a finding that had initially been missed. In others, the change may have reflected greater reliance on the AI suspicion level.

The pattern was different for confidence-only changes. In these cases, greater trust in AI over initial judgment was the dominant reason for modification. This distinction is important because a BI-RADS change prompted by an AI-highlighted finding may reflect active reassessment of the image, whereas a confidence-only change based mainly on trust in AI may reflect passive reassurance. This type of interaction is relevant to automation bias, particularly when AI output increases confidence without necessarily changing image-based reasoning.

Previous work in mammography has shown that AI-generated BI-RADS suggestions can influence radiologists’ decisions and may contribute to automation bias, including when AI recommendations are incorrect. Dratsch et al. demonstrated that AI suggestions affected reader performance across different levels of experience, emphasizing that AI output can shape interpretation rather than simply support it [21]. More broadly, automation bias has been recognized as a safety concern in clinical decision-support systems, especially when users accept algorithmic recommendations without sufficient independent verification.

In the present study, automation bias cannot be directly confirmed because the study did not evaluate the cognitive process behind each decision and did not test intentionally incorrect AI suggestions. However, the high proportion of confidence-only modifications attributed to trust in AI suggests that residents may be particularly sensitive to algorithmic reassurance. Therefore, AI should be presented to trainees as a decision-support tool that prompts targeted reassessment, rather than as an authoritative second opinion. The key educational task is to teach residents when to accept AI output, when to question it, and when to override it based on imaging findings and BI-RADS reasoning.

### 4.6. Educational Implications for Radiology Training

The findings may also have implications for radiology training, although the study was not designed to evaluate a formal educational intervention. Mammography interpretation is known to have a substantial learning curve, and residents often require repeated exposure to develop reliable diagnostic thresholds and confidence [3,4,5]. AI may support this process by drawing attention to subtle abnormalities, encouraging reassessment of uncertain cases, and providing an additional decision-support input during image interpretation.

However, the educational value of AI depends on how it is used. Passive acceptance of AI output may promote overreliance, whereas active comparison between AI suggestions and image-based reasoning may support learning. Similar effects of AI assistance on less experienced readers have been reported outside breast imaging. In ultrasound-based thyroid nodule assessment, AI support helped junior radiologists achieve diagnostic performance comparable to that of senior radiologists [22]. In addition, AI-assisted fracture detection during residency training has been shown to influence both diagnostic performance and confidence [23]. These findings support the broader idea that AI may be useful for trainees and less experienced readers, but only when integrated with critical image-based reasoning rather than used as a substitute for independent interpretation.

Therefore, AI-assisted interpretation in training settings should emphasize critical appraisal, verification of AI-suggested findings, and confidence calibration rather than simple agreement with algorithmic output.

### 4.7. Strengths and Limitations

This study has several strengths. First, it used a paired multi-reader design in which all residents evaluated the same examinations before and after AI exposure. This allowed within-reader comparison of BI-RADS classification and confidence. Second, the study focused on radiology residents, a clinically relevant population in whom AI may have both educational benefits and potential risks related to overreliance.

Third, the analysis extended beyond simple BI-RADS changes. It included diagnostic confidence, confidence-only changes, reasons for modification, expert-reference agreement, and resident-level variability. This provides a broader view of AI impact than studies focused only on diagnostic accuracy. Fourth, the study included an expert-consensus BI-RADS reference standard, allowing assessment of whether AI-assisted reassessment moved resident interpretations closer to or farther from expert interpretation. Finally, the breast-level analytic approach allowed separate evaluation of the right and left breasts within each examination, providing a more detailed assessment of resident decision-making.

Several limitations should be acknowledged. First, this was a retrospective, single-center study with a limited number of mammographic examinations. Although the number of resident-breast readings was large, these readings were clustered within the same cases and readers. Cluster-adjusted sensitivity analyses were added to address this structure, but the limited number of readers means that *p*-values should still be interpreted cautiously, and the findings should be considered exploratory.

Second, the expert-consensus BI-RADS reference standard was not equivalent to pathology-confirmed disease status. The reference standard allowed assessment of agreement with expert BI-RADS interpretation, but it did not define cancer diagnosis. As a result, the study cannot establish whether AI improved cancer detection or patient-level clinical outcomes. The expert-reference results should therefore be interpreted as changes in agreement with expert assessment rather than definitive diagnostic accuracy.

Third, the study evaluated AI-assisted reassessment rather than independent second-read performance. Residents were provided with their initial BI-RADS category and confidence score during the AI-assisted session. This design was appropriate for evaluating how AI influences reassessment of an already established initial opinion, but it introduces anchoring and cannot separate the effect of AI from repeat reading, recall, temporal learning, or willingness to revise a prior assessment. Therefore, the observed 9.7% BI-RADS modification rate may underestimate the magnitude of change that might occur in a fully independent AI-assisted reading design.

Fourth, the AI system analyzed only 2D mammography images, whereas residents interpreted both 2D mammography images and DBT datasets. Therefore, the AI output was generated from a more limited image set than that available to the readers, which should be considered when interpreting the observed influence of AI on resident reassessment. This limitation is relevant because recent reviews have shown that the performance of AI tools in breast imaging may vary depending on the algorithm, imaging system, and clinical setting. Therefore, careful validation is needed before AI results can be generalized across different clinical workflows [24,25].

Fifth, the cohort consisted of diagnostic and problem-solving mammography examinations rather than a screening population. The prevalence of suspicious BI-RADS categories and the behavior of readers in this setting may differ from screening mammography workflows. Therefore, the expert-threshold agreement estimates should not be generalized directly to screening performance.

### 4.8. Future Directions

Future studies should validate these findings in larger, prospective, multicenter cohorts including both residents and experienced radiologists. Ideally, future work should incorporate pathology, imaging follow-up, or multidisciplinary adjudication to evaluate whether AI-assisted reassessment improves cancer detection, false-positive rates, and patient-level outcomes. Studies should also assess whether structured AI training can reduce automation bias while preserving the educational benefits of AI support.

Further research should examine lesion-level concordance between AI markers, resident visual assessment, DBT findings, and expert interpretation. Such analyses could clarify whether AI-associated changes are driven by true lesion recognition or by non-specific shifts in perceived suspicion. Finally, longitudinal studies are needed to determine whether repeated AI-assisted training improves independent mammography performance over time or whether it increases dependence on algorithmic output.

## 5. Conclusions

AI-assisted reassessment measurably influenced mammography interpretation by radiology residents, affecting both BI-RADS classification and diagnostic confidence. Although BI-RADS changes occurred in a minority of readings, confidence was modified more frequently, indicating that the impact of AI extended beyond categorical classification to residents’ subjective certainty and decision-making behavior.

The effect of AI was directly related to the AI suspicion category and was more pronounced in readings with lower initial confidence. Expert-reference analysis showed modest improvement in agreement with the expert-consensus BI-RADS standard after AI-assisted reassessment, with the most clinically relevant signal observed at the BI-RADS 4+ threshold, where sensitivity increased while specificity remained stable. However, not all AI-associated changes moved interpretations closer to expert consensus, highlighting that AI can both support and misdirect less experienced readers.

## Figures and Tables

**Table 1:** Study cohort, imaging dataset, and reader characteristics.

Characteristic	Value
Mammographic examinations	112
Assessable case-breasts	223
Excluded case-breasts	1 right breast, absent after prior mastectomy
Resident-breast readings	2230
Participating residents	10
Senology experience	3–12 months
Less experienced readers	5
More experienced readers	5
Patient age range	25–81 years
BI-RADS density A	45
BI-RADS density B	43
BI-RADS density C	20
BI-RADS density D	4
Imaging available to residents	2D mammography and DBT
Imaging analyzed by AI	2D mammography only

**Table 2:** BI-RADS distribution before and after AI-assisted reassessment.

BI-RADS Category	Before AI, *n* (%)	After AI, *n* (%)	Difference
BI-RADS 1	265 (11.9%)	253 (11.3%)	−12
BI-RADS 2	1243 (55.7%)	1244 (55.8%)	+1
BI-RADS 3	386 (17.3%)	367 (16.5%)	−19
BI-RADS 4	254 (11.4%)	272 (12.2%)	+18
BI-RADS 5	82 (3.7%)	94 (4.2%)	+12

**Table 3:** BI-RADS modification and clinically relevant threshold crossings.

Outcome	*n*/N	Percentage
BI-RADS unchanged	2014/2230	90.3%
BI-RADS changed	216/2230	9.7%
Upgrade	125/2230	5.6%
Downgrade	91/2230	4.1%
Movement from <3 to ≥3	78	—
Movement from ≥3 to <3	67	—
Movement from <4 to ≥4	63	—
Movement from ≥4 to <4	33	—

**Table 4:** Diagnostic confidence modification after AI-assisted reassessment.

Outcome	*n*/N or Value	Percentage/Change
Confidence changed	429/2230	19.2%
Confidence unchanged	1801/2230	80.8%
Confidence increased	372/2230	16.7%
Confidence decreased	57/2230	2.6%
Confidence-only change	325/2230	14.6%
Any AI-associated modification	541/2230	24.3%
Confidence 4–5 before AI	1562/2230	70.0%
Confidence 4–5 after AI	1812/2230	81.3%
Mean confidence before AI	3.95	—
Mean confidence after AI	4.16	—
Mean confidence delta	+0.21	—

**Table 5:** BI-RADS and confidence modification according to AI suspicion category.

AI Suspicion Category	Total Readings	BI-RADS Change, *n* (%)	Upgrades	Downgrades	Any AI-Associated Modification, *n* (%)
No findings	1460	79 (5.4%)	12	67	279 (19.1%)
Low suspicion	70	6 (8.6%)	1	5	19 (27.1%)
Medium suspicion	370	65 (17.6%)	49	16	120 (32.4%)
High suspicion	330	66 (20.0%)	63	3	123 (37.3%)

**Table 6:** Reader-reported reasons for AI-associated modification.

Reason for Modification	BI-RADS Change, *n* (%)	Confidence-Only Change, *n* (%)	Any AI-Associated Modification, *n* (%)
AI highlighted overlooked finding	79 (36.6%)	18 (5.5%)	97 (17.9%)
Trust in AI over initial judgment	70 (32.4%)	271 (83.4%)	341 (63.0%)
Other/unclear	67 (31.0%)	36 (11.1%)	103 (19.0%)
Total	216 (100%)	325 (100%)	541 (100%)

**Table 7:** Expert-reference BI-RADS agreement before and after AI-assisted reassessment.

Metric	Before AI	After AI	Difference
Exact BI-RADS agreement, %	46.6	47.6	+1.0
Within-one-category agreement, %	88.6	90.0	+1.5
Mean absolute BI-RADS error	0.666	0.636	−0.030
Moved closer to expert-consensus reference	—	131	—
Moved farther from expert-consensus reference	—	80	—
Unchanged absolute BI-RADS error	—	2019	—

**Table 8:** Agreement with expert-consensus BI-RADS thresholds before and after AI-assisted reassessment. BI-RADS 4+ was prespecified as the primary clinical threshold. BI-RADS 3+ was evaluated as a secondary threshold. Metrics were calculated using the expert-consensus BI-RADS reference standard and reflect agreement with expert BI-RADS classification, not pathology-confirmed cancer detection.

Metric	Before AI	After AI	Difference
BI-RADS 4+ positive agreement, %	72.0	82.3	+10.3
BI-RADS 4+ negative agreement, %	93.8	93.8	+0.1
BI-RADS 4+ positive predictive agreement, %	64.3	67.5	+3.2
BI-RADS 4+ negative predictive agreement, %	95.6	97.2	+1.6
BI-RADS 4+ overall agreement, %	90.9	92.3	+1.4
BI-RADS 4+ balanced agreement, %	82.9	88.1	+5.2
BI-RADS 3+ positive agreement, %	81.3	85.6	+4.4
BI-RADS 3+ negative agreement, %	78.0	78.3	+0.3
BI-RADS 3+ overall agreement, %	78.6	79.6	+1.0

**Table 9:** Cluster-adjusted sensitivity analyses for key reader-behavior and expert-reference outcomes. Estimates account for clustering by reader and case-breast using two-way cluster-robust standard errors. Observed counts and percentages are descriptive; *p*-values and confidence intervals are from the cluster-adjusted models.

Outcome	Observed Result	Cluster-Adjusted Estimate	95% CI	*p*-Value
BI-RADS changed after AI-assisted reassessment	216/2230 (9.7%)	9.7%	7.6 to 12.3%	-
Upgrade among BI-RADS-changed readings	125/216 (57.9%)	57.9%	39.7 to 74.1%	0.398
Confidence increase among confidence-changed readings	372/429 (86.7%)	86.7%	71.0 to 94.6%	<0.001
BI-RADS 4+ classification rate	15.1% to 16.4%	OR 1.11	0.99 to 1.24	0.079
Expert BI-RADS 4+ threshold agreement	90.9% to 92.3%	OR 1.20	1.02 to 1.42	0.026
Exact BI-RADS agreement with expert reference	46.6% to 47.6%	OR 1.04	0.95 to 1.14	0.399
Mean absolute BI-RADS error	0.666 to 0.636	Difference −0.030	−0.063 to 0.003	0.077

**Table 10:** Reader-level variability in BI-RADS reassessment and expert-reference agreement.

Reader	BI-RADS Change Rate	Upgrades	Downgrades
Reader 1	16/223 (7.2%)	14	2
Reader 2	31/223 (13.9%)	23	8
Reader 3	17/223 (7.6%)	5	12
Reader 4	28/223 (12.6%)	19	9
Reader 5	12/223 (5.4%)	5	7
Reader 6	25/223 (11.2%)	20	5
Reader 7	30/223 (13.5%)	7	23
Reader 8	18/223 (8.1%)	16	2
Reader 9	11/223 (4.9%)	8	3
Reader 10	28/223 (12.6%)	8	20

## Data Availability

The raw data supporting the conclusions of this article will be made available by the authors on request.

## Abbreviations

The following abbreviations are used in this manuscript:

AI	Artificial Intelligence
BI-RADS	Breast Imaging Reporting and Data System
DBT	Digital Breast Tomosynthesis
